# Infantile hypertrophic pyloric stenosis with unusual presentations in Sagamu, Nigeria: a case report and review of the literature

**DOI:** 10.11604/pamj.2016.24.114.8847

**Published:** 2016-06-02

**Authors:** Tinuade Adetutu Ogunlesi, Opeyemi Temitola Kuponiyi, Collins Chigbundu Nwokoro, Ibukunolu Olufemi Ogundele, Gbenga Fakunle Abe, Olusoga Babatunde Ogunfowora

**Affiliations:** 1 Department of Paediatrics, Olabisi Onabanjo University Teaching Hospital, Sagamu, Nigeria; 2 Department of Surgery (Paediatric Surgical Unit), Olabisi Onabanjo University Teaching Hospital, Sagamu, Nigeria

**Keywords:** Abdominal mass, pyloromyotomy, gastric outlet obstruction, pyloric stenosis, newborn, vomiting

## Abstract

A 24-day old female Nigerian neonate presented with protracted vomiting, fever and dehydration but without palpable abdominal tumour or visible gastric peristalsis. There was no derangement of serum electrolytes. The initial working diagnosis was Late-Onset Sepsis but abdominal ultrasonography showed features consistent with the diagnosis of IHPS. This case report highlights the atypical presentation of this surgical condition and the need to investigate cases of protracted vomiting in the newborn with at least, ultrasonography to minimize complications and reduce the risk of mortality in a resource-poor setting.

## Introduction

Vomiting, in the newborn, frequently connotes physiologic events such as feed regurgitation or poor positioning after over-feeding and less commonly, follows serious illnesses such as asphyxia, sepsis, necrotizing enterocolitis, in-born errors of metabolism or upper gastrointestinal obstructive lesions [1]. Therefore, innocuous as it seems, feed regurgitation or vomiting may portend dangers in the newborn and unravelling the cause may be a challenge for practitioners.

Infantile hypertrophic pyloric stenosis (IHPS) is one of the surgical causes of protracted vomiting in early infancy. This condition is characterized by abnormal thickening of the muscular wall of the pylorus hence it is the commonest cause of gastric outlet obstruction in infancy. The exact aetiology of IHPS is unclear but the roles of genetic predispositions and prenatal or postnatal exposure to drugs such as the macrolides and nalidixic acid are known. The condition is not a common finding in clinical practice as earlier reported in The Gambia [2] where no case was recorded in a review of newborn surgical conditions over a two-year period. Therefore, cases of IPHS may be easily missed in clinical practice.

The classical presentation of IHPS includes persistent copious non-bilous vomiting (which may become projectile), visible intestinal peristalsis, palpable epigastric tumour, constipation, failure to thrive despite sucking hungrily, dehydration and electrolytes derangements such as hypokalaemia and metabolic alkalosis. However, inconsistencies in the pattern of the aforementioned clinical features in IHPS have been reported [3] and that makes strong clinical suspicion imperative to the prevention of missed or delayed diagnosis and increased risk of complications. Therefore, this case is reported to highlight the protean clinical and laboratory characteristics of this relatively uncommon condition which may be associated with mortality [3]. This report is intended to improve clinical suspicion of IHPS when practitioners are confronted with newborns and early infants with protracted vomiting.

## Patient and observation

A 24-day old female neonate presented at the Children's Emergency Room of the Olabisi Onabanjo University Teaching Hospital, Sagamu, southwest Nigeria on referral from a General Hospital on account of persistent vomiting and high grade continuous fever of five days duration. The vomiting was described as projectile, post-prandial, non-bilous and of large volume. The frequency of vomiting was 4 to 5 times daily. Although the infant had not opened the bowel for two days prior to the onset of the illness, there was no abdominal distension at any point. Apart from scanty urinary output, the baby presented with no other symptoms.

This infant was the first child of her mother - a 30-year old second wife, known epileptic. The pregnancy was booked for antenatal care in a secondary health facility at about 22 weeks of gestation. The prenatal and perinatal periods were uneventful and the mother could only recall taking routinely prescribed haematinics and anti-convulsants in pregnancy. From birth, the infant had been exclusively breastfed and adequately immunized.

At presentation, the infant weighed 2kg, was febrile (axillary temperature - 400C), but was not pale or icteric. However, she was conscious and severely dehydrated but had no other systemic abnormalities. The initial diagnosis was Late-Onset Neonatal Sepsis with severe dehydration to exclude upper intestinal obstruction. The baby was admitted into the Neonatal Ward and the haematocrit, random blood glucose and serum electrolytes, urea and creatinine were within normal. Dehydration was appropriately corrected using intravenous fluids Normal Saline. Subsequently, the baby received maintenance intravenous 8%Dextrose in 5th Saline, intravenous antibiotics (ceftriaxone and gentamicin) and was placed on nil per os.

**Figure 1:**
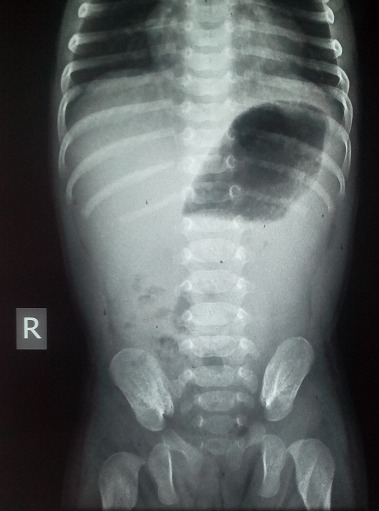
Plain abdominal X-Ray (erect) showing single huge air bubble in the stomach

The vomiting persisted despite management, but without fever. At this point, the possibility of IHPS was entertained and test-feeding was done but no epigastric mass was palpable. However, abdominal ultrasound scan revealed distended stomach with food and fluid residual, active peristaltic waves without emptying, thickened muscular pyloric canal measuring 7.5cm in length and 0.74cm in thickness. The typical “doughnut-shaped” appearance of the gastric shadow in transverse abdominal scan was seen. These findings are consistent with the diagnosis of Infantile Hypertrophic Pyloric Stenosis. Plain abdominal X-Ray in the erect position showed gross gastric dilatation (Figure 1).

**Figure 2:**
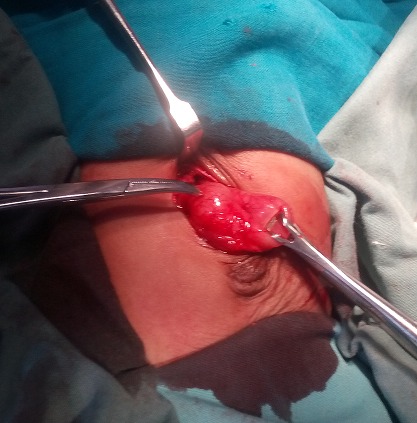
Intra-operative picture showing artery forceps pointing at the pyloric tumour

Gastric decompression with an open-ended nasogastric tube was commenced, maintenance intravenous fluid was continued with strict monitoring of fluid input and output with subsequent paediatric surgical intervention. Pyloromyotomy (Figure 2) was done and the baby did well post operatively. She was discharged home on the 5th post-operative day without complications.

## Discussion

This case report aims at highlighting the diagnostic challenges which may be encountered in IHPS. The admission diagnosis was Late-Onset Neonatal Sepsis based on the history of protracted vomiting, fever and dehydration. These are known features of septicaemia in the newborn. The persistence of vomiting despite intravenous antibiotic therapy prompted the managing team to consider an obstructive upper gastrointestinal condition. This implies the need to consider both medical and surgical causes when young infants present early in life with protracted vomiting.

The infantile hypertrophic pyloric stenosis, previously known as congenital pyloric stenosis, is characterized by postnatal hypertrophy of the circular and longitudinal muscles of the pylorus resulting in a pyloric swelling which blends into the gastric antrum at the proximal end but ends abruptly at the duodenum distally. The pyloric canal is elongated, thickened and narrow with eventual gastric outlet obstruction and secondary hypertrophy of the stomach. In a review of cases of IHPS, increased pyloric canal length, diameter and thickness were reported in 98%, 87% and 60% of cases [4].

Despite the varying incidence across different regions, the condition is less common in blacks and female children, same groups to which the index case belongs [5]. However, the index case was the first child of the family as frequently reported [3]. The details of probable etiological factors could not be explored due to poor maternal mental state and possible limited information at the disposal of the informant. The specific anti-convulsant drug the mother received in pregnancy could not be identified. It is also unknown if any of the commonly used anti-convulsants have been associated with the development of IHPS in the babies of mothers with epilepsy.

The age of the index baby at presentation agreed with the frequently reported four to six weeks [3]. The acute course of the disease seen in term infants, typified by this neonate was not in tandem with the usual clinical and biochemical findings in cases of infantile hypertrophic pyloric stenosis. Despite the usual symptoms of vomiting and dehydration with fever, the well described intestinal peristaltic wave was not seen and no pyloric mass was palpable in this infant as previously reported that only less than 30% of IHPS cases had palpable tumours [3, 4]. Therefore, relying almost exclusively on the palpation of epigastric masses will result in missed and delayed diagnosis. This observation was similar to the findings in the case reported by Egri-Okwaji *et al* in Lagos, Nigeria [6]. In the latter case, the characteristic clinical signs were also absent and the diagnosis was made by imaging studies and confirmed intra-operatively [6]. This is in contrast to majority of cases (60-80%) in which these clinical manifestations are present [7].

The typical serum electrolyte derangements in IHPS include hypokalaemia, hyponatraemia and hypochloraemic metabolic alkalosis [8]. This pattern of derangements had earlier been reported in the majority of babies studied in Zaria, northern Nigeria [9] and other parts of the world [8]. In these earlier reports, electrolytes derangements were not observed among infants who presented early. This may explain the normal values of the serum electrolytes recorded in the index infant as the physiologic compensatory mechanisms would maintain homeostasis in the early stage of the disease.

Abdominal ultrasound was useful in making a clinical diagnosis in the index baby. Indeed, this imaging method has been reported to make accurate diagnosis in 98% of cases [4]. This observation agreed with the findings in a study which ascribed decreased detection of the olive-shaped abdominal mass in IHPS by palpation to increased availability of ultrasonography and early use of this imaging study in suspected cases [10]. Ultrasound is recommended as first line investigation in babies with unexplained protracted vomiting because it is readily available, cheap and cost-effective.

## Conclusion

In IHPS, clinical diagnosis may be difficult when it is strictly based on the clinical and laboratory features. In addition, normal values of serum electrolytes do not preclude the diagnosis in IHPS. Therefore, being the commonest surgical cause of non-billous vomiting in infancy, a high index of suspicion is important for early diagnosis, prompt intervention and avoidance of mortality.

## References

[1] Barr DGD, Hendrickse RG, Barr DGD, Matthews TS (1991). Systematic Neonatology. Paediatrics in the Tropics.

[2] Bickler SW, Sanno-Duanda B (2000). Epidemiology of paediatric surgical admissions to a government referral hospital in the Gambia. Bull World Health Org..

[3] Chalya PL, Manyama M, Kayange NM, Mabula JB, Massenga A (2015). Infantile hypertrophic pyloric stenosis at a tertiary care hospital in Tanzania: a surgical experience with 102 patients over a 5-year period. BMC Res Notes..

[4] Khan AA, Yousaf MA, Ashraf M (2014). Role of ultrasonography in early diagnosis of Infantile Hypertrophic Pyloric Stenosis. J Ayub Med Coll Abbottabad..

[5] To T, Wajja A, Wales PW, Langer JC (2005). Population demographic indicators associated with incidence of pyloric stenosis. Arch Pediatr Adolesc Med..

[6] Egri - Okwaji MTC, Njokanma OF, Uba CC, Osuoji RI, Fajolu IB (2005). Unusual presentation of Infantile Hypertrophic Pyloric Stenosis in a ten-week old infant. Niger J Paediatr..

[7] Markowitz RI (2014). Olive without a cause: the story of infantile hypertrophic pyloric stenosis. Pediatr Radiol..

[8] Walker-Smith JA, Mittal SK, Hendrickse RG, Hendrickse RG, Barr DGD, Matthews TS (1991). Systematic Neonatology. Paediatrics in the Tropics.

[9] Nnamadu PT (1992). Alterations in serum electrolytes in congenital hypertrophic stenosis: a study in Nigerian children. Ann Trop Paediatr..

[10] Bakal U, Sarac M, Aydin M, Tarter T, Kazoz A (2015). Recent changes in the features of hypertrophic pyloric stenosis. Pediatr Int.

